# Altered white matter microstructural integrity in patients with postherpetic neuralgia: a combined DTI and DTI-NODDI study

**DOI:** 10.3389/fnins.2025.1552961

**Published:** 2025-02-18

**Authors:** Wei Qian, Xiaopei Xu, Ying Wu, Lina Yu, Chao Wang, Min Yan, Risheng Yu

**Affiliations:** ^1^Department of Radiology, the Second Affiliated Hospital, Zhejiang University School of Medicine, Zhejiang, China; ^2^Department of Anesthesiology, the Second Affiliated Hospital, Zhejiang University School of Medicine, Hangzhou, China

**Keywords:** herpes zoster, postherpetic neuralgia, diffusion tensor imaging, neurite orientation dispersion and density imaging, white matter microstructure

## Abstract

**Background:**

Postherpetic neuralgia (PHN) is a debilitating condition resulting from herpes zoster infection, characterized by persistent pain that significantly impacts quality of life. This study aimed to investigate the white matter microstructural alterations associated with PHN and to assess the relationship between diffusion metrics and clinical symptoms.

**Methods:**

A total of 29 patients with PHN, 28 patients recovering from herpes zoster (RHZ), and 27 healthy controls (HC) were recruited, and clinical assessments were obtained to evaluate pain intensity and psychological distress. Diffusion tensor imaging (DTI) data was collected, followed by analysis of diffusion and neurite orientation dispersion and density imaging (NODDI) metrics. Statistical analyses included ANOVA to compare groups and Pearson correlation coefficients to assess relationships between imaging metrics and clinical outcomes.

**Results:**

PHN patients exhibited significantly altered white matter integrity, specifically in neurite density index (NDI) and orientation dispersion index, compared to both RHZ patients and HC. Significant correlations were also found between altered imaging metrics and clinical assessments of pain and emotional distress, with lower fractional anisotropy (FA) and NDI associated with higher pain scores and psychological symptoms.

**Conclusion:**

Our study highlights significant microstructural changes in white matter tracts in patients with PHN, indicating compromised neural integrity that correlates with increased pain perception and emotional distress. NODDI demonstrated superior sensitivity in detecting these alterations compared to traditional DTI metrics, underscoring its potential for enhancing diagnostic and therapeutic approaches in managing chronic pain conditions like PHN.

## Introduction

Herpes zoster (HZ), induced by the varicella zoster virus (VZV), predominantly presents as clustered herpetic lesions, erythema, and neuropathic pain localized to the affected dermis (Cohen, 2013; Harbecke et al., 2020). This condition is self-limiting, with acute pain subsiding as the herpetic lesions resolve. However, recuperation from herpes zoster (RHZ) can be complicated by the emergence of postherpetic neuralgia (PHN), a prevalent and persistent complication affecting up to 30% of individuals over 60 years (Johnson, 2010; Johnson and Rice, 2014). PHN is defined by enduring pain within the affected dermatome, persisting for at least 3 months post-HZ rash clearance. This pain is frequently characterized by a burning, stabbing, or shooting sensation, and could have widespread and devastating effects on patients’ quality-of-life in the acute and chronic phases, affecting their physical and psychological health, as well as their ability to continue normal daily and social activities (Katz et al., 2004). Psychological impacts, such as anxiety and depression, are also frequently observed in PHN patients, contributing to feelings of helplessness, frustration, and social withdrawal, further exacerbating the physical symptoms (Schmader, 1999). However, PHN is incorrectly perceived by many clinicians to be a mild and readily treatable disease, while treatment options actually remain inadequate. Given its high prevalence, chronicity, and challenging management, PHN represents a significant public health issue among the elderly, associated with substantial morbidity, including depression, anxiety, and sleep disruptions (Saguil et al., 2017). The etiology of PHN is multifaceted, implicating factors such as age, herpetic involvement, pain intensity, and dermal lesions (Niemeyer et al., 2024). While PHN is traditionally attributed to neural disturbances in primary afferent fibers and the dorsal horn, accumulating evidence suggests a role for supraspinal modifications in perpetuating the pain experience. Recent neuroimaging studies (Liu et al., 2022; Wu et al., 2022) have revealed structural alterations in the brain’s white matter, particularly in regions implicated in sensory processing and pain regulation. These insights underscore PHN as a complex neuropathic disorder, involving both peripheral and central nervous system dysfunctions.

Diagnosing and managing HZ and PHN remain complex due to the intricate microstructural changes in both peripheral and central nervous systems (Wu et al., 2022). These alterations in both peripheral and central neural pathways contribute to persistent and debilitating pain (Li Y. et al., 2024), yet standard diagnostic tools often fail to detect the subtle disruptions in nerve fiber integrity and neural connectivity that are critical in PHN pathophysiology (Yu, 2016). Clinicians must rely heavily on patient-reported symptoms and the clinical presentation of HZ, which can be highly variable and subjective (Saguil et al., 2017). This subjectivity complicates treatment, necessitating individualized approaches based on each patient’s experience. To address these challenges, advances in imaging technologies, such as diffusion tensor imaging (DTI), are needed to capture microstructural and functional changes more accurately (Dai et al., 2020; Jiang et al., 2022; Wu et al., 2022). Significant decreases in white matter integrity were observed in the left inferior fronto-occipital fasciculus, indicating disrupted microstructure associated with chronic pain in PHN (Jiang et al., 2022). Additionally, further research into the pathophysiological mechanisms of PHN is essential for developing targeted therapies that go beyond symptomatic relief, ultimately improving patient outcomes.

Neurite Orientation Dispersion and Density Imaging (NODDI) (Zhang et al., 2012) is an advanced MRI technique that provides a detailed examination of brain microstructure beyond the capabilities of traditional DTI. While DTI measures diffusion characteristics like fractional anisotropy (FA), it often struggles with complex microstructural interpretations due to limitations like free water contamination (Grussu et al., 2015; Reddy and Rathi, 2016; By et al., 2017). NODDI addresses these challenges by utilizing a multicompartment model to estimate neurite density and orientation dispersion thus allows for the separation of free water signals from tissue signals, offering a clearer and more accurate depiction of microstructural organization (Tariq et al., 2016). The NODDI-DTI model further enhances this approach by allowing for the extraction of biophysical parameters from standard single-shell DTI data (Edwards et al., 2017). While the NODDI-DTI model simplifies the original NODDI framework, enabling time-efficient analysis, it holds promise for advancing our understanding of neural pathologies, offering both a nuanced interpretation of existing datasets and a streamlined method for estimating biophysical parameters from smaller diffusion datasets (Stein et al., 2024). This innovation allows researchers to leverage existing datasets for deeper insights while maintaining efficiency in data collection and analysis, particularly in complex conditions like PHN.

In this study, we sought to determine the effectiveness of NODDI-DTI as an *in vivo* imaging marker for detecting pathology related to PHN and to demonstrate the specificity of this technique over standard DTI measures, thus we included three groups of participants: patients with PHN, recuperation from herpes zoster (RHZ) patients, and healthy controls (HC). Our primary hypothesis was that patients with PHN would exhibit impaired microstructural integrity compared to those who had fully recovered from HZ and the healthy controls. Furthermore, we anticipated that NODDI-DTI metrics would correlate more strongly with clinical symptoms, as indicated by clinical parameters, than traditional DTI metrics. We also hypothesized that NODDI-DTI would demonstrate superior sensitivity and specificity in identifying microstructural changes associated with PHN.

## Materials and methods

### Participants

This study was conducted in accordance with the ethical standards set forth by the Ethics Committee of the Second Affiliated Hospital of Zhejiang University, School of Medicine, which approved all study procedures. Written informed consent was obtained from each participant prior to enrollment. A total of 29 patients with PHN (mean age ± SD, 62.90 ± 6.76 years), 28 RHZ patients (mean age ± SD, 61.71 ± 6.57 years), and 27 age-matched healthy controls (HC) (mean age ± SD, 59.48 ± 7.30 years) were recruited for this study. PHN patients were diagnosed by a clinician according to the criteria established by the International Association for the Study of Pain (IASP), which required a pain intensity of ≥4 on the Visual Analog Scale (VAS; 0 = no pain, 10 = worst pain imaginable) and a disease duration of at least 3 months following the onset of acute HZ. All PHN patients were receiving a stable dosage of medication for at least 2 weeks prior to the study. In contrast, RHZ patients were diagnosed based on self-reported pain intensity scores of <4/10 on the VAS, a duration of at least 3 months post-acute HZ, and no ongoing medical treatment for HZ. Exclusion criteria for all participants included: (1) left-handedness; (2) special forms of HZ (including ear, eye, visceral, or asymptomatic HZ); (3) history of ongoing acute or chronic pain conditions (such as headaches, toothaches, arthritis, or cervical/lumbar spondylopathy); (4) psychiatric or neurological disorders, including mood disorders, epilepsy, or a history of head injury; (5) serious health conditions, such as severe cardiovascular diseases or renal insufficiency with unstable hemodynamics; (6) any other ongoing medications (except for those prescribed for PHN treatment); and (7) any contraindications for MRI.

### Clinical assessments

All clinical assessments were conducted 1 h prior to brain scanning. Each participant completed the short-form McGill Pain Questionnaire (MPQ), which includes 12 sensory descriptors and 4 affective descriptors to evaluate sensory and affective pain scores, respectively. Additionally, participants assessed their pain using the Visual Analog Scale (VAS), the Present Pain Intensity (PPI) scale, and the ID Pain score for neuropathic pain assessment. Depression and anxiety levels were measured using the Hamilton Depression Scale (HAMD) and the Hamilton Anxiety Scale (HAMA). Participants also completed the Positive Affect Negative Affect Schedule (PANAS), which assesses positive and negative affect through its two components. Furthermore, the Medical Outcomes Study 36-item Short Form Survey (SF-36) was administered to evaluate health-related quality of life, covering various domains such as physical functioning, role-physical, bodily pain, general health, vitality, social functioning, role-emotional, mental health, and reported health transition.

### Image acquisition

All scans were performed on a 3.0 Tesla MRI scanner (Discovery MR750, GE Healthcare) equipped with an eight-channel head coil in the department of radiology, the second affiliated hospital, zhejiang university school of medicine. During the scanning, the head of each subject was stabilized with a restraining foam pad, and scanning noise was attenuated using earplugs. High-resolution structural T1-weighted images were acquired using a fast spoiled gradient recalled sequence using the following parameters: repetition time (TR)/echo time (TE) = 7.4/3.0 ms, flip angle = 11°, field of view (FOV) = 260 × 260 mm^2^, matrix size = 256 × 256, slice thickness = 1.2 mm, 196 continuous sagittal slices. Single shell diffusion images were acquired using spin-echo planar imaging sequence with 30 gradient directions for a non-zero *b* value (*b* value = 1,000 s/mm^2^). The detailed parameters were as follows: TR/TE = 8000/81 ms; flip angle = 90°; FOV = 256 × 256 mm; matrix = 128 × 128; slice thickness = 2 mm; slice gap = 0 mm; 67 interleaved axial slices.

### Image processing

DTI images were processed using the FMRIB Software Library (FSL) version 6.0.5.2.^1^ Initial steps involved correction for eddy-current distortions and involuntary movements; raw DTI volumes were linearly registered and resampled to align with the first b0 volume. The diffusion tensor was then fitted to each voxel using FSL’s DTIFit, enabling the calculation of FA maps.

To derive additional metrics related to neurite characteristics, we applied NODDI-DTI (Edwards et al., 2017), a single-shell diffusion approximation of NODDI (Zhang et al., 2012). This approach allowed us to calculate the neurite density index (NDI, or intra-cellular volume fraction, ICVF) and the orientation dispersion index (ODI) from the DTI data using a custom MATLAB script. Following the guides from Edwards et al. (2017), NODDI-DTI exclusively utilized FA and MD maps as inputs for these calculations.

The FA data were then transformed to the FMRIB58-FA template in MNI152 standard space through symmetric image normalization, utilizing the ANTs package. With the aid of FSL’s tract-based spatial statistics (TBSS) toolbox, we generated a mean FA image from all participant scans within this standardized space. This resulted in a common white matter skeleton representing shared tracts across the group, with a threshold of 0.2 applied to exclude gray matter and partial volume effects. The aligned FA volume was projected onto the skeleton by interpolating FA values from the nearest relevant tract center. We conducted careful visual inspections of the output images and the thresholded skeleton to ensure their accuracy. Similar nonlinear warping and projection techniques were subsequently applied to the NDI and ODI maps.

For detailed atlas-based analyses of white matter tracts, we utilized the JHU ICBM-DTI-81 digital white matter atlas,^2^ which operates in the MNI152 standard space. This atlas encompasses 48 distinct brain regions, categorized into several types, including brainstem, commissural, projection, and association fibers. The regions and their corresponding abbreviations are presented in Table 1. Binary mask images for each tract were applied to the individual skeletonized maps, which had already been registered to the MNI152 standard space. Regional values were calculated by averaging the voxel values within the designated JHU white matter tract masks for each participant across the generated DTI and NODDI-DTI parametric maps.

**Table 1:** The 48 distinct regions and their corresponding abbreviations of the JHU ICBM-DTI-81 digital white matter atlas.

Region	Abbreviation	Category
Middle cerebellar peduncle	MCP	Brainstem
Pontine crossing tract	PCT	Brainstem
Genu of the corpus callosum	gCC	Commissural
Body of the corpus callosum	bCC	Commissural
Splenium of the corpus callosum	sCC	Commissural
Fornix	FX	Association
Right corticospinal tract	CST-R	Brainstem
Left corticospinal tract	CST-L	Brainstem
Right medial lemniscus	ML-R	Brainstem
Left medial lemniscus	ML-L	Brainstem
Right inferior cerebellar peduncle	ICP-R	Brainstem
Left inferior cerebellar peduncle	ICP-L	Brainstem
Right superior cerebellar peduncle	SCP-R	Brainstem
Left superior cerebellar peduncle	SCP-L	Brainstem
Right cerebral peduncle	CP-R	Brainstem
Left cerebral peduncle	CP-L	Brainstem
Right anterior limb of the internal capsule	ALIC-R	Projection
Left anterior limb of the internal capsule	ALIC-L	Projection
Right posterior limb of the internal capsule	PLIC-R	Projection
Left posterior limb of the internal capsule	PLIC-L	Projection
Right retrolenticular part of the internal capsule	RIC-R	Projection
Left retrolenticular part of the internal capsule	RIC-L	Projection
Right anterior corona radiata	ACR-R	Projection
Left anterior corona radiata	ACR-L	Projection
Right superior corona radiata	SCR-R	Projection
Left superior corona radiata	SCR-L	Projection
Right posterior corona radiata	PCR-R	Projection
Left posterior corona radiata	PCR-L	Projection
Right posterior thalamic radiation	PTR-R	Projection
Left posterior thalamic radiation	PTR-L	Projection
Right sagittal stratum	SS-R	Association
Left sagittal stratum	SS-L	Association
Right external capsule	EC-R	Association
Left external capsule	EC-L	Association
Right cingulum (cingulate gyrus)	CgC-R	Association
Left cingulum (cingulate gyrus)	CgC-L	Association
Right cingulum (hippocampus)	CgH-R	Association
Left cingulum (hippocampus)	CgH-L	Association
Right fornix (cres) / stria terminalis	FX/ST-R	Association
Left fornix (cres) / stria terminalis	FX/ST-L	Association
Right superior longitudinal fasciculus	SLF-R	Association
Left superior longitudinal fasciculus	SLF-L	Association
Right superior fronto-occipital fasciculus	SFOF-R	Association
Left superior fronto-occipital fasciculus	SFOF-L	Association
Right uncinate fasciculus	UF-R	Association
Left uncinate fasciculus	UF-L	Association
Right tapetum	TAP-R	Commissural
Left tapetum	TAP-L	Commissural

### Statistical analysis

Demographic and clinical variables among the three groups (PHN, RHZ, and HC) were statistically analyzed using SPSS 24.0 software (SPSS, Inc., Chicago, IL, United States). Continuous variables are presented as mean ± SD and were evaluated for normality using the Shapiro–Wilk test. For variables that demonstrated normal distribution, intergroup differences were assessed using one-way ANOVA. For categorical variables, differences were analyzed using chi-squared tests or Fisher’s exact tests as appropriate. A significance level of *p* < 0.05 was established for all analyses.

To examine DTI and NODDI-DTI related parameters across the three groups, we employed one-way ANCOVA, incorporating age and sex as covariates. To account for multiple comparisons, *p*-values were adjusted using the false discovery rate (FDR) correction. *Post hoc* analyses were conducted to evaluate pairwise differences among the groups.

In addition, to explore the association of DTI and NODDI-DTI parameters with clinical variables—such as MPQ, VAS, PPI, ID Pain, HAMD, HAMA, PANAS, and SF-36 scores—partial correlation analyses were performed. These analyses controlled for age, sex, and education years. Given that some pain-related clinical variables (MPQ, VAS, PPI, and ID Pain) were not available for the HC group, these were exclusively analyzed within the RHZ and PHN groups. Conversely, other clinical variables (HAMD, HAMA, PANAS, and SF-36 scores) were included in the partial correlation analyses across all groups. A significance threshold of *p* < 0.05 was applied, with FDR correction used to adjust for multiple comparisons across clinical measures.

## Results

### Demographics

Detailed demographics are shown in Table 2. A total of 84 patients were recruited for this study, comprising 29 patients with PHN (mean age ± SD, 62.90 ± 6.76 years), 28 patients with RHZ (mean age ± SD, 61.71 ± 6.57 years), and 27 HCs (mean age ± SD, 59.48 ± 7.30 years). There were no statistically significant differences between the groups in terms of gender (PHN: males/females, 20/9; RHZ: males/females, 13/15; HC: males/females, 14/13; *p* = 0.201) and age (*p* = 0.177). In terms of clinical characteristics, PHN patients exhibited significantly higher pain scores when compared to RHZ patients, as measured by the VAS (*p* < 0.001), MPQ sensory/affective (p < 0.001), ID Pain scores (p < 0.001), and PPI (p < 0.001), whereas pain-related scores in the HC group were unavailable. Compared with RHZ patients and HCs, PHN patients showed significantly elevated scores on the HAMD (*p* < 0.001) and HAMA (*p* < 0.001). Furthermore, PHN patients reported higher PANAS negative score (*p* < 0.001), lower PANAS positive score (*p* < 0.001) and lower SF-36 (*p* < 0.001) compared to both RHZ patients and HCs. In comparison, RHZ patients displayed slightly higher pain scores (including VAS and PPI, *p* < 0.001), higher HAMD (*p* = 0.024), lower PANAS positive score (*p* < 0.001) and SF-36 scores (*p* < 0.001) relative to HCs.

**Table 2:** Detailed demographic and clinical information of the participants in this study.

	PHN (*n* = 29)	RHZ (*n* = 28)	HC (*n* = 27)	*p* value
Gender (males/females)	20/9	13/15	14/13	0.201
Age (mean ± SD, years)	62.90 ± 6.76	61.71 ± 6.57	59.48 ± 7.3	0.177
Pain VAS (mean ± SD)	5.38 ± 1.15	0.86 ± 0.93	/	<0.001
MPQ sensory (mean ± SD)	7.21 ± 3.14	0.64 ± 0.73	/	<0.001
MPQ affective (mean ± SD)	2.10 ± 1.08	0.14 ± 0.36	/	<0.001
PPI (mean ± SD)	2.48 ± 0.63	0.54 ± 0.51	/	<0.001
ID Pain (mean ± SD)	2.48 ± 1.12	0.32 ± 0.48	/	<0.001
HAMD (mean ± SD)	6.93 ± 2.79 ^a,c^	2.68 ± 2.57	1.19 ± 1.69	<0.001^*^
HAMA (mean ± SD)	4.55 ± 2.34 ^a,c^	1.57 ± 1.62	0.78 ± 1.28	<0.001^*^
PANAS positive (mean ± SD)	12.21 ± 1.66 ^a,c^	14.86 ± 2.46 ^b^	23.89 ± 2.44	<0.001^*^
PANAS negative (mean ± SD)	16.76 ± 3.16 ^a,c^	10.75 ± 1.40	10.74 ± 1.31	<0.001^*^
SF-36 (mean ± SD)	112.92 ± 7.49 ^a,c^	127.51 ± 5.67 ^b^	136.03 ± 3.58	<0.001^*^

PHN, postherpetic neuralgia; RHZ recuperation from herpes zoster; HC, healthy controls; VAS, visual analogous scale; MPQ, McGill pain questionnaire; PPI, present pain intensity; HAMD, Hamilton Depression Scale; HAMA, Hamilton Anxiety Scale; PANAS, positive affect negative affect score; SF-36, 36-item short form from health survey. *Indicates a significant difference between PHN, RHZ, and HC; a indicates a significant difference between HC and PHN; b indicates a significant difference between HC and RHZ; and c indicates a significant difference between PHN and RHZ.

### Regional tract-specific analysis

ROI analyses using ANCOVA, accounting for age and sex, revealed widespread differences in DTI and NODDI metrics across various white matter regions. Notably, a greater number of significant differences were observed in NODDI metrics compared to DTI metrics.

**Figure 1:**
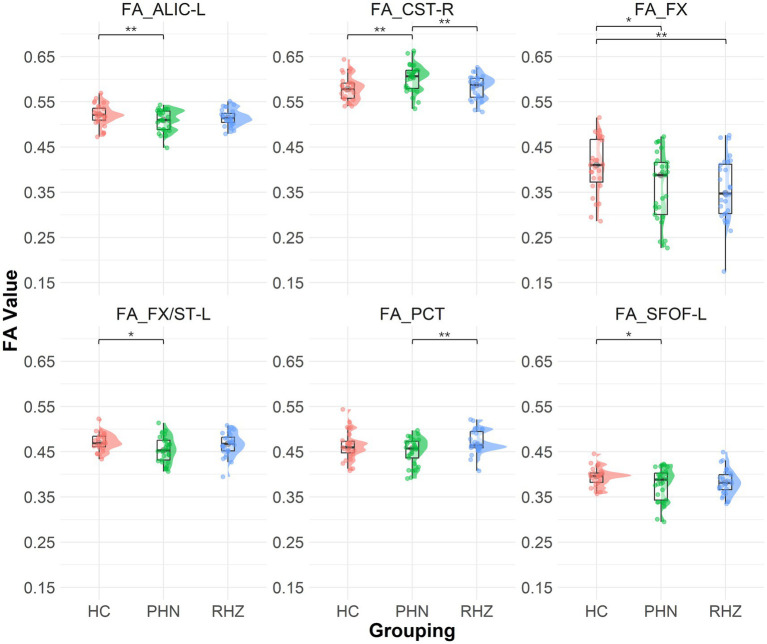
The Raincloud plot visualizes the distribution of fractional anisotropy (FA) values for selected white matter tracts that demonstrate significant differences between patient groups (PHN, postherpetic neuralgia; RHZ recuperation from herpes zoster; HC, healthy controls). The y-axis represents the FA values, while the x-axis categorizes the data by patient grouping. The plot integrates a density cloud to illustrate data distribution and jittered raw data points to represent individual observations. Each facet corresponds to a specific tract with significant differences, including the fornix column and body of the fornix (FX), left anterior limb of the internal capsule (ALIC-L), left fornix crescent (FX/ST-L), left superior fronto-occipital fasciculus (SFOF-L), right corticospinal tract (CST-R), and pontine crossing tract (PCT). Significant pairwise differences are marked by asterisks (*p < 0.05; **p < 0.01).

The DTI analysis indicated significant differences in FA values across groups (Figure 1). When compared to controls, the PHN group exhibited lower FA values in the FX (*p* = 0.014), ALIC-L (*p* = 0.004), FX/ST-L (*p* = 0.019), and SFOF-L (*p* = 0.041). Conversely, the CST-R in the PHN group showed a significant increase in FA (*p* = 0.005). When comparing the PHN group to the RHZ group, the CST-R also displayed a significant FA increase (*p* = 0.007), while the PCT in the PHN group demonstrated a significant decrease in FA (*p* = 0.009). Additionally, the RHZ group showed a significant decrease in FA in the FX compared to controls (*p* = 0.005).

**Figure 2:**
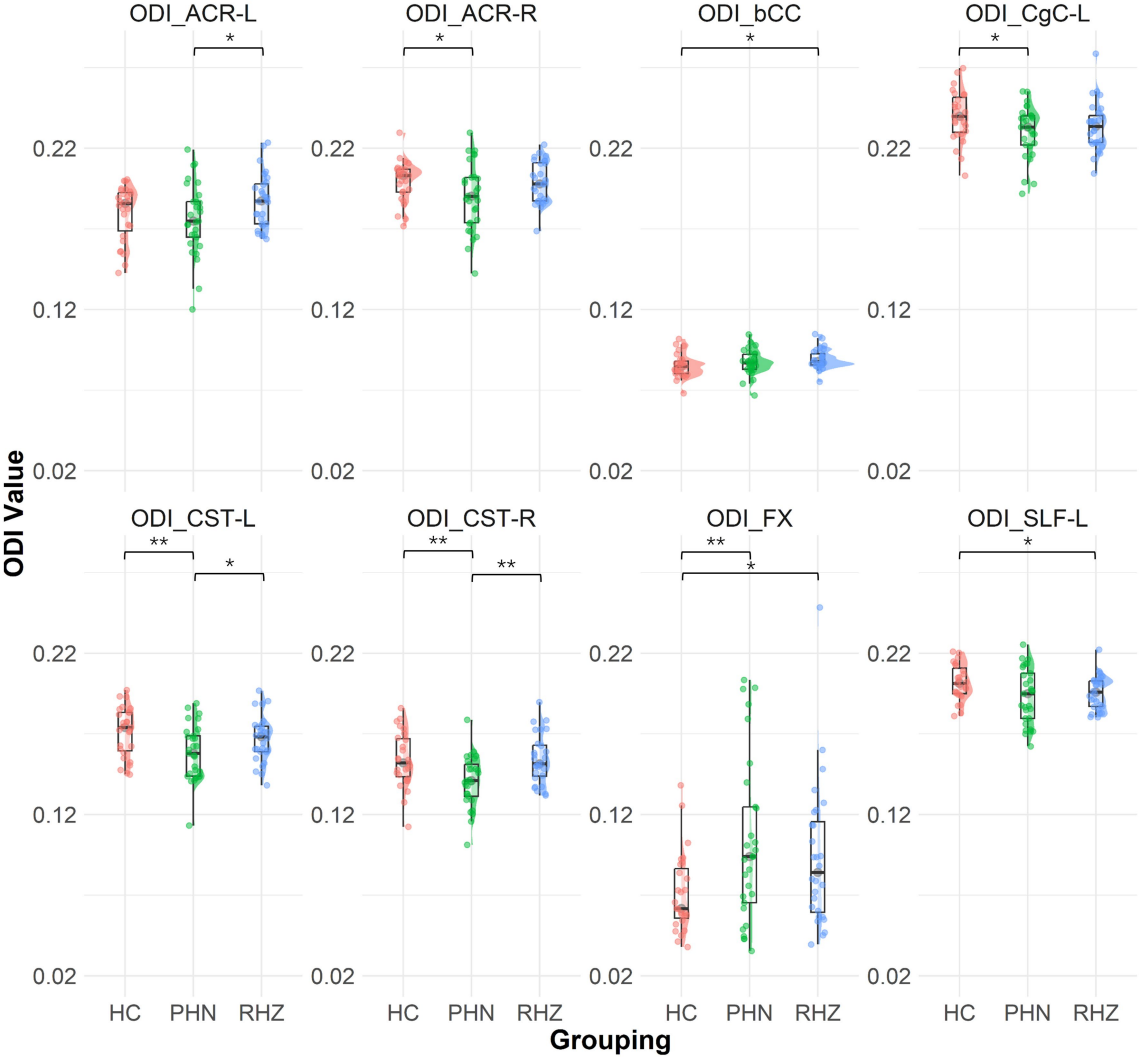
The Raincloud plot visualizes the distribution of orientation dispersion index (ODI) values for selected white matter tracts that demonstrate significant differences between patient groups (PHN, postherpetic neuralgia; RHZ recuperation from herpes zoster; HC, healthy controls). The y-axis represents the ODI values, while the x-axis categorizes the data by patient grouping. The plot integrates a density cloud to illustrate data distribution and jittered raw data points to represent individual observations. Each facet corresponds to a specific tract with significant differences, including fornix column and body of the fornix (FX), left and right corticospinal tract (CST-L, CST-R), left cingulum (cingulate gyrus) (CgC-L), left superior longitudinal fasciculus (SLF-L), left and right anterior corona radiata (ACR-L, ACR-R), and body of the corpus callosum (bCC). Significant pairwise differences are marked by asterisks (*p < 0.05; **p < 0.01).

The NODDI analysis revealed significant differences in NDI and ODI (Figure 2) across groups. The RHZ group had a notably lower NDI in the FX compared to controls (*p* = 0.002). In terms of ODI, the PHN group exhibited a significantly elevated ODI in the FX when compared to controls (*p* = 0.006). Furthermore, the PHN cohort showed reduced ODI values in the CST-R and CST-L (*p* = 0.004 for both), ACR-R (*p* = 0.039), CgC-L (*p* = 0.028), and SLF-L (*p* = 0.030). The RHZ group demonstrated lower ODI in the SLF-L (*p* = 0.036) but higher ODI in the bCC (*p* = 0.027) and FX (*p* = 0.027) when compared to controls. Additionally, the RHZ group exhibited higher ODI in the CST-R (*p* = 0.002), CST-L (*p* = 0.045), and ACR-L (*p* = 0.035) compared to the PHN cohort.

### Association with clinical symptoms

**Figure 3:**
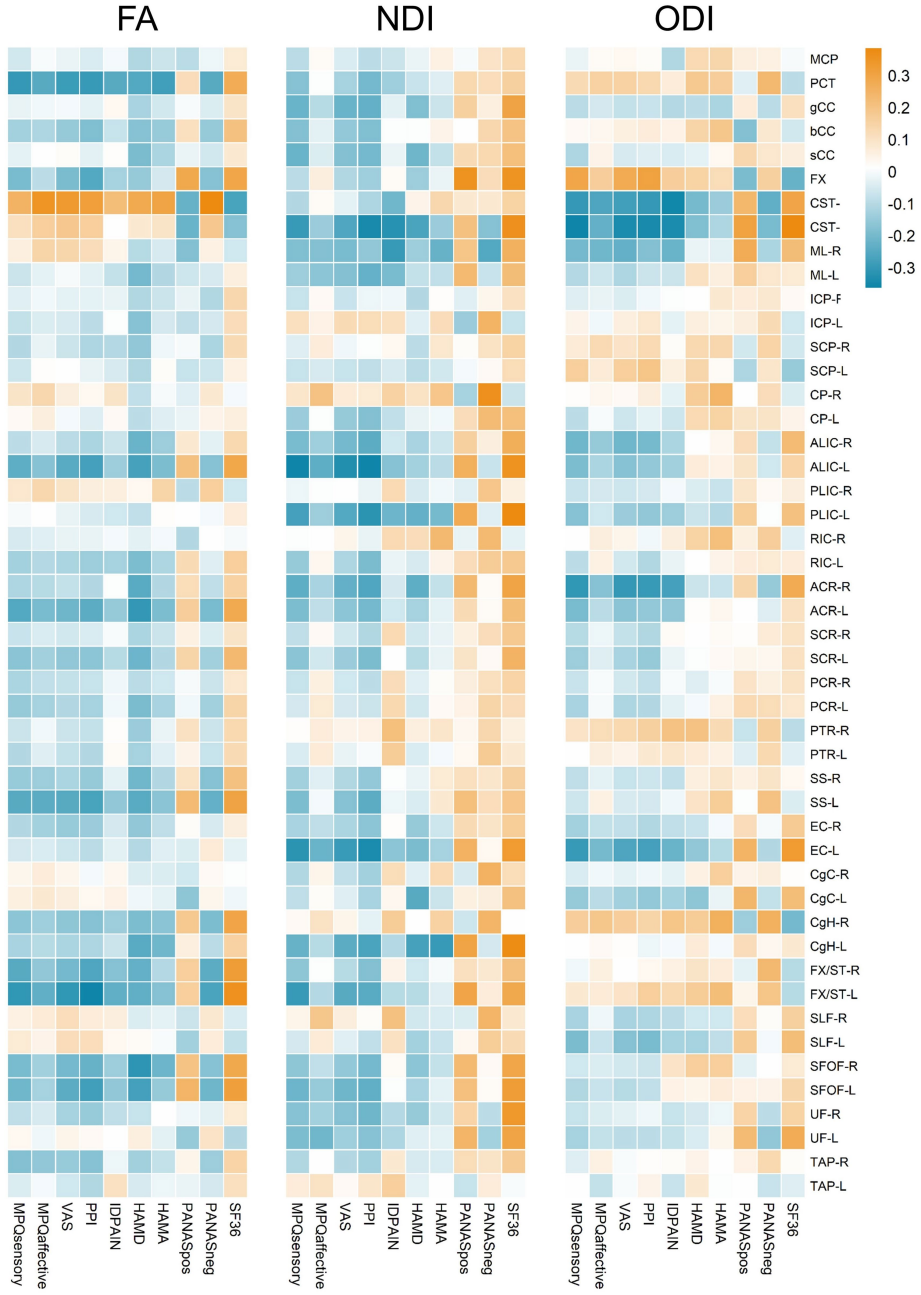
Heatmap representation of correlation coefficients between FA, NDI, ODI values of white matter tracts and multiple clinical assessments. Correlation coefficients are color-coded as shown on the top of the figure. The color gradient, ranging from blue to orange, indicates the strength and direction of the correlations, with blue representing negative correlations and orange indicating positive correlations.

When examining the relationship between clinical symptoms and FA, significant correlations were found in various tracts, and detailed results are shown in Figure 3. Notably, The FA of the PCT was negatively associated with MPQ sensory (*r* = −0.270, *p* = 0.015), VAS (*r* = −0.257, *p* = 0.020), PPI (*r* = −0.267, *p* = 0.016), ID pain score (*r* = −0.225, *p* = 0.044), HAMA (*r* = −0.270, *p* = 0.015), PANAS negative score (*r* = −0.230, *p* = 0.039), and positively associated with SF-36 (*r* = 0.237, *p* = 0.033). The FA of the CST-R showed positive associations with MPQ affective (*r* = 0.319, *p* = 0.004), VAS (*r* = 0.290, *p* = 0.009), PPI (*r* = 0.269, *p* = 0.015), HAMD (*r* = 0.325, *p* = 0.003), HAMA (*r* = 0.349, *p* = 0.001), PANAS negative score (*r* = 0.379, *p* < 0.001), and negatively associated with SF-36 (*r* = −0.274, *p* = 0.013). For the FX/ST-L, the FA was negatively correlated with MPQ sensory (*r* = −0.243, *p* = 0.029), VAS (*r* = −0.236, *p* = 0.034), PPI (*r* = −0.304, *p* = 0.006), and positively associated with SF-36 (*r* = 0.274, *p* = 0.013). The NDI of the CST-L was negatively associated with MPQ sensory (*r* = −0.264, *p* = 0.017), VAS (*r* = −0.225, *p* = 0.044), PPI (*r* = −0.277, *p* = 0.012), and ID pain score (*r* = −0.282, *p* = 0.011). The ODI of the PCT was positively associated with HAMA (*r* = 0.263, *p* = 0.018) and PANAS negative score (*r* = 0.270, *p* = 0.015). The ODI of the CST-R showed negative correlations with MPQ (sensory: *r* = −0.261, *p* = 0.018; affective: *r* = −0.246, *p* = 0.028), VAS (*r* = −0.266, *p* = 0.016), PPI (*r* = −0.288, *p* = 0.009), ID pain score (*r* = −0.312, *p* = 0.005), HAMD (*r* = −0.300, *p* = 0.006), PANAS negative score (*r* = −0.279, *p* = 0.012), and positively associated with SF-36 (*r* = 0.240, *p* = 0.031). Lastly, the ODI of the CST-L was negatively correlated with MPQ (sensory: *r* = −0.284, *p* = 0.010; affective: *r* = −0.235, *p* = 0.035), VAS (*r* = −0.312, *p* = 0.005), PPI (*r* = −0.330, *p* = 0.003), ID pain score (*r* = −0.244, *p* = 0.028), PANAS negative score (*r* = −0.247, *p* = 0.026), and positively associated with SF-36 (*r* = 0.298, *p* = 0.007). The ODI of the ACR-R exhibited negative correlations with MPQ (sensory: *r* = −0.325, *p* = 0.003; affective: *r* = −0.248, *p* = 0.026), VAS (*r* = −0.279, *p* = 0.012), PPI (*r* = −0.297, *p* = 0.007), ID pain score (*r* = −0.297, *p* = 0.007), PANAS negative score (*r* = −0.230, *p* = 0.039), and positively with SF-36 (*r* = 0.280, *p* = 0.011).

## Discussion

In this study, we investigated the microstructural alterations associated with PHN through the application of DTI and NODDI. Our findings revealed that patients with PHN exhibited significantly lower FA in key white matter tracts, including the fornix and the anterior limb of the internal capsule, compared to healthy controls, indicating compromised neural integrity. Conversely, RHZ patients demonstrated relatively elevated FA, indicative of more robust neural coherence. Moreover, the NODDI metrics demonstrated enhanced sensitivity in detecting alterations in neurite density and orientation dispersion, highlighting their potential as specific markers for PHN-related pathophysiology of chronic pain. Furthermore, we observed significant correlations between diminished imaging parameters—particularly within the posterior cingulate tract—and clinical assessments of pain and psychological distress, underscoring the relationship between microstructural integrity and patient-reported outcomes.

White matter, composed of myelinated axons, is essential for facilitating signal transmission between neurons and coordinating the activities of various brain regions. Recent studies (Liu et al., 2019, 2022) have highlighted alterations in cortical thickness and gray matter volume in individuals with PHN compared to healthy controls. Prolonged pain can induce significant plasticity in both peripheral and central nociceptive systems, leading to structural changes at molecular, synaptic, and cellular levels (Kuner, 2010). Utilizing DTI, researchers can reconstruct white matter pathways and assess microstructural changes to fiber tracts through metrics like FA, which serves as an indicator of myelin integrity. Lower FA values typically suggest more diffuse or damaged fiber tracts, reflecting the degeneration of myelinated axons (Beaulieu, 2002). Animal models (Laemmle et al., 2019) have shown that the reactivation of the latent HZ virus leads to axonal degeneration and damage to satellite glial cells and Schwann cells, resulting in myelin loss. Furthermore, chronic pain can influence the function and chemical properties of primary sensory neurons and dorsal horn neurons (Woolf and Salter, 2000), thereby affecting neuroplasticity and the overall anatomy of the brain. Building upon the findings derived from DTI, NODDI offers enhanced insights into neurite density and orientation dispersion, thereby facilitating a more nuanced understanding of underlying neurobiological processes. NODDI combines diffusion signals with tissue-specific features through a biophysical model, allowing for the quantification of neurite morphology in both white matter and gray matter (Zhang et al., 2012). It enhances the specificity of diffusion imaging by approximating each voxel into three distinct compartments: the intra-neurite compartment, which reflects neurite density (NDI); and the extra-neurite compartment, which assesses the orientation consistency of neurites (ODI). The ability of NODDI to measure both NDI and ODI provides critical insights into the integrity of neural structures, making it more sensitive to detecting changes compared to conventional DTI methods (Edwards et al., 2017). Specifically, lower NDI values indicate potential loss or damage of neurites, while higher ODI values can signify increased crossing of neurites. Consequently, the application of NODDI in our analysis allows for a more detailed exploration of the microstructural alterations observed in our study, as reflected in the significant differences in NDI and ODI across groups.

This study represents the first application of NODDI to investigate microstructural alterations in patients with PHN, highlighting increased sensitivity in detecting more specific characterization of WM microstructural changes compared with conventional DTI metrics. Our findings reveal that the RHZ group exhibited a significantly lower NDI in the FX compared to controls, suggesting a reduction in the density of neurites and potentially indicating compromised neural architecture in this region, which may contribute to difficulties in pain management and emotional regulation often reported by patients with chronic pain conditions (Histological metrics confirm microstructural characteristics of NODDI indices in multiple sclerosis spinal cord, 2015). Conversely, the elevated ODI observed in the PHN group within the FX indicates increased neurite orientation variability. From a neurobiological perspective, this heightened dispersion may reflect maladaptive plasticity, where the brain undergoes structural changes in response to prolonged pain. Increased ODI can also suggest a disorganization of neural pathways (Li Z. et al., 2024), which may interfere with the efficient transmission of pain-related signals and contribute to the heightened pain experience characteristic of PHN. This finding aligns with literature indicating that chronic pain can lead to maladaptive changes in the brain’s structure and function (Barroso et al., 2021; De Ridder et al., 2021; Mainero et al., 2011). Additionally, the PHN cohort showed reduced ODI values across several tracts, including the CST, ACR, CgC, and SLF. These tracts are integral to sensory and motor functions, and alterations in their microstructure may correlate with impaired pain modulation and sensory processing (Noseda and Burstein, 2013). The RHZ group, on the other hand, displayed distinct patterns, with lower ODI in the SLF but higher ODI in the bCC compared to controls. This suggests variations in neural connectivity and structural organization that require further investigation. The body of the corpus callosum is essential for interhemispheric communication, and alterations in this region may impact the integration of sensory information and cognitive processing related to pain (Mei et al., 2023). Given that the corpus callosum consists of parallel fibers, the impact of white matter fiber looseness alone is insufficient to yield significant results (Huang et al., 2022), and only the NODDI derived metrics could effectively detect changes in the corpus callosum. NODDI demonstrated more widespread changes compared to the parameters obtained from DTI, suggesting that NODDI is more sensitive to the influences of PHN. The application of NODDI provides critical insights into the specific microstructural changes associated with PHN. By differentiating between NDI and ODI, we gain a clearer understanding of the neurobiological alterations in patients with chronic pain. These findings underscore the potential of NODDI as a powerful tool for elucidating the complex relationships between neural plasticity, pain perception, and cognitive-emotional processing, ultimately paving the way for more targeted diagnostic and therapeutic strategies in managing chronic pain conditions.

Our findings regarding microstructural integrity in patients with PHN reveal significant alterations across several other key brain regions, such as FX, ALIC and SFOF, each providing insight into the neurobiological mechanisms underlying this chronic pain condition. Notably, the decreased FA we found in FX, which connects the hippocampus to various brain structures, may indicate its impact in memory processing and emotional regulation (Choi et al., 2021) in PHN as compared to controls. Our results are in line with a previous study (Liu et al., 2018) which found reduced FA in the internal capsule, external capsule, and FX in patients with trigeminal neuralgia. In PHN patients, compromised white matter integrity of the FX may lead to difficulties in managing emotional responses to pain, as emotional distress often exacerbates pain perception (Bushnell et al., 2013; Hayes et al., 2017). This disruption in cognitive and emotional functioning creates a feedback loop, where poor emotional regulation contributes to heightened pain experiences. The relationship between emotional processing and cognitive functioning finds further reflection in the ALIC, which serves as a key conduit for transmitting information between the frontal cortex and subcortical structures (Mithani et al., 2020). The ALIC, like the fornix, is integral to both emotional and cognitive regulation, facilitating executive functions such as decision-making and response modulation (Mithani et al., 2020; Zhang et al., 2021). Binshabaib et al. (2019) found that ALIC may predict the development of chronic pain after back injury, which supports the idea that ALIC can modulate learned affective behavioral responses (Machado et al., 2009) thus would potentially serve as a target substrate for deep brain stimulation (Plow et al., 2013) aimed at mitigating pain-related disability. The impaired microstructural integrity we observed in ALIC indicates that patients with PHN may experience deficits in these areas, complicating their ability to adaptively manage pain. Conversely, we found significantly increased FA in CST of PHN when compared to controls. The CST is crucial for motor control and contributes significantly to the modulation of pain (Kristo et al., 2013; Kim et al., 2018). The integrity of the CST is essential for executing voluntary movements and adaptive responses to pain, and changes in this tract among PHN patients may reflect a maladaptive plasticity wherein chronic pain alters typical neural pathways, leading to increased sensitivity and potential motor dysfunction (Mansour et al., 2013). The CST’s role in both motor and pain modulation underscores the interconnectedness of emotional and cognitive processes; disruptions in emotional regulation via the ALIC may lead to decreased motor control, further complicating the pain experience.

In comparing the PHN and RHZ groups, we observed a significant increase in FA in the CST-R in the PHN group, which may reflect adaptive neuroplastic changes in response to persistent pain (Rayhan et al., 2013; Li Z. et al., 2024). The degree of both axonal and myelin changes was greater in PHN than in RHZ, supporting the idea that white matter microstructural damage is more severe in PHN. These findings share similarities with the previously discussed compromised tract integrity in PHN, further elucidating the complex relationship between chronic pain and microstructural changes in the brain. Indeed, increased responsiveness in the primary motor cortex and CST was repeatedly demonstrated in migraineurs when using transcranial magnetic stimulation (Brighina et al., 2011; Mainero et al., 2011). This increase in FA could suggest enhanced connectivity; however, it also raises concerns regarding maladaptive plasticity, as indicated by Mansour et al. (2013), who noted that chronic pain can lead to heightened sensitivity and potential motor dysfunction. Furthermore, the RHZ group demonstrated a significant decrease in FA in FX, mirroring changes seen in the PHN group; the FX, crucial for connecting the hippocampus with limbic structures (Choi et al., 2021), underscores the role of emotional and cognitive processing in pain experiences. Collectively, these findings reveal that microstructural alterations in a network of brain regions contribute to a cumulative decline in cognitive, emotional, and motor functioning, thereby perpetuating the chronic pain cycle in PHN patients, highlighting the need to consider both axonal and myelin-related changes in understanding the underlying neural mechanisms.

In addition to the group differences we previously identified, we further discovered correlations between diffusion-related parameters and clinical measures, suggesting that these microstructural changes may play a crucial role in sensory pain perception and emotional regulation. Specifically, the negative correlations between FA in the PCT and clinical measures such as MPQ sensory, VAS, and HAMA suggest that reduced microstructural integrity in PCT may exacerbate sensory pain and mood disturbances, highlighting its critical role in PHN. Chronic pain has substantial adverse effects on emotional functioning, and emotional distress is common in PHN (Katz et al., 2004). Jiang et al. (2023) found that the effective connection from the lateral amygdala to the anterior cingulate cortex was reduced in PHN patients, and the functional connection of both were positively correlated with HAMA and HAMD scores, suggesting that chronic pain in PHN patients promotes the development of anxiety and depression. Cortical thickness in the right anterior cingulate gyrus and medial prefrontal cortex, linked to anxiety and depression, was correlated with the duration of HZ, suggesting that changes in these areas related to negative emotions are influenced by disease duration (Liu et al., 2022). As the entry point for cortical inputs to the cerebellum via the middle cerebellar peduncle (Baumann et al., 2015), PCT plays a crucial role in modulating pain perception (Bocci et al., 2015), and also implies the involvement of the cerebellum in processing nociceptive inputs. Alterations in the integrity of the PCT, as reflected by FA values, might influence how pain and anxiety are experienced and reported by patients. Moreover, the positive association we observed between the PCT’s ODI and HAMA also suggested its involvement in anxiety-related responses, supporting studies (Ghazi Sherbaf et al., 2018; Li et al., 2020; Fan et al., 2022; Kang et al., 2023) that have identified PCT’s contribution to emotional and depressive symptoms. In the CST, the observed relationship where higher FA correlates with increased affective pain and mood disturbances is consistent with its known role in modulating pain and emotional responses (Lam et al., 2019). This finding is supported by literature indicating that the CST is involved in both motor and sensory pathways (Kristo et al., 2013; Kim et al., 2018), which are often disrupted in chronic pain syndromes(Baliki et al., 2012). Conversely, the negative correlation of ODI with sensory and affective pain highlights the structural adaptations that may contribute to pain resilience, corroborating studies that suggest decreased ODI may indicate more organized neural pathways, potentially reducing sensory disturbance. This finding supports the previous speculation of Jones et al. (2013) that increased FA could stem from decreased crossing of axons, higher axon density or from higher density of glia cells such as oligodendrocytes. Furthermore, NDI findings in the CST-L highlight its significant role in sensory pain modulation (Frezel et al., 2023), aligning with studies (Tolner et al., 2019; Li Z. et al., 2024; Shibata and Ishiyama, 2024) that have demonstrated how changes in neurite density can affect pain transmission. Furthermore, the negative ODI correlations in regions like ACR-R point to broader structural disorganization contributing to the multifaceted symptomatology of PHN and RHZ. These findings indicate not only the pain modulation and sensory pain perception are related to microstructural alterations, but also emotion regulation is essential to white matter integrity in PHN patients.

Our study bears several limitations that warrant consideration. Primarily, the relatively small sample size in all three groups may contribute to less reliable results, as it potentially limits the generalizability and statistical power of our findings. The patients with PHN in our cohort reported moderate pain intensity, which may partly explain the lack of significant differences in some diffusion metrics. Additionally, the absence of longitudinal data restricts our ability to observe ongoing microstructural white matter changes associated with PHN pain over time. Intra-individual differences were also not accounted for, which could influence the results, particularly given the small sample size. Furthermore, the limited PHN course duration in our study, due to time constraints, may not fully capture the subtle differences in white matter microstructural changes between the acute and sequelae stages of HZ. Moreover, it remains unclear whether these observed changes in white matter are primary contributors to PHN pain or secondary effects resulting from functional changes, a common limitation in imaging studies of this nature. Insights from mapping intrinsic brain connectivity networks, as highlighted in recent studies (Zhang et al., 2024; Cheng et al., 2025), suggest that understanding these networks could provide a mechanistic framework for human behavior, which may also be applicable to the understanding of pain perception in PHN. Future research should aim for larger, more diverse sample sizes and incorporate longitudinal follow-ups to better understand the temporal progression of brain structural changes in PHN patients and their potential reversibility with treatment.

## Conclusion

In conclusion, our study reveals significant microstructural changes in multiple white matter tracts in patients with PHN. Moreover, these key alterations were linked to pain and emotional distress. These changes were strongly correlated with heightened pain perception and emotional distress, suggesting a link between neural integrity and clinical symptoms. NODDI demonstrated superior sensitivity in identifying these alterations, indicating its potential for enhancing diagnostic and therapeutic approaches in managing chronic pain conditions like PHN.

## Data Availability

The original contributions presented in the study are included in the article/supplementary material, further inquiries can be directed to the corresponding authors.

## Footnotes

^1^ https://fsl.fmrib.ox.ac.uk/fsl

^2^ http://cmrm.med.jhmi.edu
